# Extrapolation of toxic indices among test objects

**DOI:** 10.2478/v10102-010-0054-7

**Published:** 2010-12

**Authors:** Miloň Tichý, Marián Rucki, Zdeněk Roth, Iveta Hanzlíková, Alena Vlková, Jana Tumová, Rút Uzlová

**Affiliations:** National Institute of Public Health, Šrobárova 48, 10042 Praha 10, Czech Republic

**Keywords:** QAAR, QSAR, extrapolation, acute toxicity, oligochaeta, fish, ciliate, hepatocytes

## Abstract

Oligochaeta *Tubifex tubifex*, fish fathead minnow (*Pimephales promelas*), hepatocytes isolated from rat liver and ciliated protozoan are absolutely different organisms and yet their acute toxicity indices correlate. Correlation equations for special effects were developed for a large heterogeneous series of compounds (QSAR, quantitative structure-activity relationships). Knowing those correlation equations and their statistic evaluation, one can extrapolate the toxic indices. The reason is that a common physicochemical property governs the biological effect, namely the partition coefficient between two unmissible phases, simulated generally by n-octanol and water. This may mean that the transport of chemicals towards a target is responsible for the magnitude of the effect, rather than reactivity, as one would assume suppose.

## Introduction

The hazard of new chemicals in both environmental and human risk assessment attracts attention in all chemical safety programmes. To detect it is the responsibility of their manufacturers or distributors. The manufacturers, regulatory bodies or industrial research, are looking for an effective, rapid and inexpensive way of estimating a potential harm defined by various programmes of chemical safety. The standard tests of toxicity involve often the use of experimental animals. The concept of 3Rs (Replacement, Reduction and Refinement) (Russell & Burch 1959) call however for reduction of animals for experiments. Thus, alternative methods of toxicity testing have been searched. The alternative methods involve both *in vitro* techniques (*e.g.* Botham 2004; Artz *et al*., 2008; Schaefer et al., 2008) and *in silico* procedures (QSAR – quantitative structure-activity relationships) (Jaworska *et al*., 2003; Cronin et al*.*, 2003) and their validation to allow a regulatory acceptance of their results (Wold 1991, Tichy *et al*., 2005, Tichy & Rucki 2009).

A set of comparative studies have been published as QAAR (quantitative activity-activity relationship). The data on various aquatic organisms, as *Poecilia reticulate*, *Pimephales promelas,Tetrahymena pyriformis, Vibrio fischeri* or *Tubifex tubifex* (Schultz *et al*., 1986; Lipnick 1985; Bearden & Schultz 1997, 1998; Kaiser 1993; Cronin *et al*., 1991; Tichy *et al*., 2008), or on activities of rat hepatocytes (Tichy *et al*., 2010) were used for these studies. To discover factors of toxicity variation among various species, acute toxicity data of many aquatic species and chemicals were analyzed by pattern recognition techniques (Vaal *et al*., 1997). Patterns in species sensitivity were found to be more diffuse because only part of the variance in species sensitivity could be explained. Most variations were due to differences in the toxicity of compounds and not to intrinsic differences among species.

Data on toxicity measured with several aquatic organisms such as *Tetrahymena pyriformis, Daphnia magna* or *Pimephales promelas* correlate between each other. The extrapolation indicates that the accuracy of the estimated toxic activity was unaffected by the extrapolation. The estimated toxic activity was comparable with the activity measured with individual bioassays, at least for noncovalent acting chemicals (Dimitrov *et al*., 2000).

In the present study, extrapolation of acute toxicity indices measured with oligochaeta *Tubifex tubifex*, fathead minnow (*Pimephales promelas*) and indices of metabolic disorder measured with primary rat hepatocytes are discussed. The data on EC_50_ inhibiting movement of the oligochaeta *Tubifex* in 3 mins (Tichy *et al*., 2007), EC_50_ of cell viability and EC_50_ influencing metabolic function of primary rat hepatocytes, ureogenesis (Tichy *et al*., 2010), EC_50_ causing effect on fish *Pimephales promelas* in 96 hours (Kaiser *et al*., 1997) were used.

## Material and methods

### Chemicals

The chemicals used were of pro-analysis grade purity, purchased from Fluka and Aldrich (Buchs, Switzerland) or Lancaster (Johnson Matthey, Ward Hill, Maryland, USA). Standard chemical manganese chloride was obtained from Merck (Darmstadt, Germany).

### Experimental organisms

*Tubifex tubifex* also called sludge worm or sewage worm, is a species of tubificid segmented worm that inhabits sediments of rivers, lakes and ponds on several continents. These worms ingest bacteria from the sediments and absorb molecules through their body wall.They can survive in areas heavily polluted with organic matter that almost no other organisms endure. They can survive with very little oxygen.

*Pimephales promelas*, fathead minnow, is a species of temperate fresh water fish belonging to the cyprinid family. The natural geographic range extends throughout much of North America including Canada. It is a golden or xanthic strain, known as the rosy-red minnow, and is a very common feeder fish sold in the United States. In its wild original form, the fathead minnow is generally dull olive-gray in appearance with a dusky stripe extending along the back and side and with a lighter belly. It inhabits muddy pools of headwaters, creeks and small rivers, ponds and lakes. It tolerates unsuitable turbid, hot or poorly oxygenated waters. EPA guidelines outline its use for the evaluation of acute and chronic toxicity of samples or chemicals in vertebrate animals.

Hepatocytes are cells of livers forming liver tissue and are responsible for a majority of processes in the liver. They include a large amount of mitochondria, a developed Golgi complex, endoplasmatic reticulum and other organelles They store glycogen, vitamins D, B12 and others. Their surface is smooth and the cells are stuck closely together. Their regeneration ability is high.

### Statistical analysis

The QSARs were generated using a linear regression procedure providing the correlation coefficient (r), standard errors of the slope and of intercept and the residual standard deviation (SD) of the estimate (Origin 2003). The statistics of predictive parameters were performed as recommended (Eriksson *et al*., 2003) and the log P-based QSARs were validated in three ways (Tichy *et al*., 2008).

### Toxicity indices

The acute toxicity indices were taken from different published sources: *Tubifex tubifex* (Tt) (Tichy *et al*., 2007), fish *Pimephales promelas* (Pp) (Kaiser *et al*., 1997) and primary rat hepatocytes ure – ureogenesis, via – viability (Tichy *et al*., 2010).

### Physicochemical descriptor

The logarithm of n-octanol-water partition coefficient, log P was taken from tables by Hansch *et al*. (1995).

## Results

The primary data used are published in the relevant papers cited with the correlation equations (Tichy *et al*., 2008, Tichy *et al*., 2010).

The correlation equations involving log P were found as follows:

$$\begin{array}{l} log E{\text{C}}_{50}(ure)=-0.840(\pm 0.054) log P-0.33(\pm 0.065)\\ n=15;r=0.975; SD=0.188 \end{array}$$

$$\begin{array}{l} log E{\text{C}}_{50}(via)=-0.778(\pm 0.053)log P-0.199(\pm 0.065)\\ n=15; r=0.971; SD=0.187 \end{array}$$

$$\begin{array}{l} log E{\text{C}}_{50}(Tt)=-0.809(\pm 0.035)log P-0.495(\pm 0.060)\\ n=82; r=0.931; SD=0.315 \end{array}$$

log EC50
(
Pp)
=−0.848 log P−0.179n=30; r=0.977; SD=0.159

log EC50
(
Pp)
=−1.027 log P−1.107n=16; r=0.899; SD=0.546

The correlations of EC_50_ of various test objects with log EC_50_(Tt) result in (Tichy *et al*., 2010)

$$\begin{array}{l} log E{\text{C}}_{50}(ure)=0.941(\pm 0.083) log E{\text{C}}_{50}(Tt)-0.124(\pm 0.107)\\ n=12; r=0.963; SD=0.236 \end{array}$$

$$\begin{array}{l} log E{\text{C}}_{50}(via)=0.910(\pm 0.0.061) log E{\text{C}}_{50}(Tt)-0.019(\pm 0.079)\\ n=12; r=0.978; SD=0.177 \end{array}$$

*or*

$$\begin{array}{l} log E{\text{C}}_{50}(via)=0.907(\pm 0.0.056) log E{\text{C}}_{50}(ure)+0.063(\pm 0.071)\\ n=15; r=0.976; SD=0.171 \end{array}$$

These are just a few examples. It is possible to receive other intercorrelations. The predictivity and robustness of equations/models given above was checked by cross validation techniques. The squared correlation coefficient r^2^ of the equation log EC_50_(via) vs. log EC_50_(Tt) changed from 0.956 to cross-validated squared correlation coefficient q^2^=0.934, the predictivity index PRESS (Predictive Residual Sum of Squares) from 0.968 to 2.041 and PRESD (Predictive Standard Deviation) from 0.284 to 0.412. The parameters of the equation for ureogenesis changed also insignificantly: r^2^=0.928 to q^2^=0.824, PRESS from 2.102 to 4.999 and PRESD from 0.419 to 0.645. The models are highly predictive and robust.

The predictive indices for the statistical evaluation of models were defined as (Eriksson *et al*., 2003):

PRESS = σ(exp – calcd)^2^, sum of differences squared between experimental and estimated (calculated) data,

PRESD = (PRESS/n)^1/2^, the second root of PRESS divided by the number of chemicals in the set.

## Discussion

The presence of log P in all log EC_50_'s allows cross correlations. This is true at least of a series of chemicals of baseline toxicity (Schultz *et al*., 1994, Lipnick 1990). The correlation with log P makes it possible to use the rapid *Tubifex* assay (Tichy *et al*., 2007). Despite phylogenetic differences between the test objects and dramatic differences in test protocols, the QSARs for nonpolar narcosis are extremely consistent (Schultz *et al*., 1990). The concept of mechanism-based QSARs is successful even for freshwater benthic organisms as *Tubifex tubifex* and for acute exposure lasting minutes (Tichy *et al*. 2008).

The log P – based QSAR described for predicting acute toxicity index EC_50_(Tt) measured with oligochaeta *Tubifex tubifex* can be recommended as an ecotoxicological model for predicting harmful effects of chemical compounds in ecological systems.

The fact that this assay can be used for determination of acute toxicity indices measured with higher organisms and under longer exposure periods supports the idea that the transport process is a prevailing step limiting the magnitude of an effect. The role of toxicokinetics can be the reason that results obtained with the *Tubifex* assay correlate with results obtained with higher organisms, and especially, including longer exposures (Tichy *et al*., 2007), when the involvement of biotransformation is considered (Veith *et al*., 1989; Lipnick *et al*., 1985). Although mechanisms of action of reactive chemical compounds were described (Veith *et al*., 1989; Lipnick *et al* 1985), toxicokinetics may also play a role in the differences among biological test objects as well.
